# Clinical Lectures

**Published:** 1874-12

**Authors:** 


*Clinical Lectures on Various Important Diseases; being a Collection of The Clinical Lectures delivered in the Medical Wards of Mercy Hospital, Chicago*, By N. S. Davis, M. D., etc., edited by Frank H. Davis, M. D. Philadelphia: Henry C. Lea, 1874. Buffalo: T. Butler & Son.

This little book containing a little over two hundred and eighty pages, is composed of the reports of clinical lectures in a modified form, which have appeared from time to time in the *Chicago Medical Examiner*. These lectures were delivered by N. S. Davis, M. D., Prof. of the Principles and Practice of Medicine in the Chicago Medical College, in the Wards of Mercy Hospital, Chicago. Made up from reports of the lectures by various writers, they necessarily lack that uniformity of style which would otherwise be expected.

These lectures are twenty in number, and embrace a wide scope in the field of general practice. The following partial list will give the reader some idea of the topics embraced, continued fever, periodical fever, scarlitina, pulmonary diseases, intestinal and uterine irritation, summer complaint of children, cardiac disease, nervous affections, cutaneous diseases, and mania a potu.

The lectures are delivered in a plain and concise manner, and were evidently intended to instruct the students of the College rather than to make any display of learning or rhetoric. In some respects they may differ from the opinions of practitioners, but on the whole they will be found to be in accordance with the generally received opinions of the day.

